# Physiological Signaling Functions of Reactive Oxygen Species in Stem Cells: From Flies to Man

**DOI:** 10.3389/fcell.2021.714370

**Published:** 2021-08-06

**Authors:** Sergey A. Sinenko, Tatiana Yu. Starkova, Andrey A. Kuzmin, Alexey N. Tomilin

**Affiliations:** Institute of Cytology, Russian Academy of Sciences, St. Petersburg, Russia

**Keywords:** mitochondria, reactive oxygen species, drosophila, embryonic stem cells, induced pluripotent stem cells, electron transport chain, hematopoiesis, HMGB1

## Abstract

Reactive oxygen species (ROS), superoxide anion and hydrogen peroxide, are generated as byproducts of oxidative phosphorylation in the mitochondria or via cell signaling-induced NADPH oxidases in the cytosol. In the recent two decades, a plethora of studies established that elevated ROS levels generated by oxidative eustress are crucial physiological mediators of many cellular and developmental processes. In this review, we discuss the mechanisms of ROS generation and regulation, current understanding of ROS functions in the maintenance of adult and embryonic stem cells, as well as in the process of cell reprogramming to a pluripotent state. Recently discovered cell-non-autonomous ROS functions mediated by growth factors are crucial for controlling cell differentiation and cellular immune response in *Drosophila*. Importantly, many physiological functions of ROS discovered in *Drosophila* may allow for deciphering and understanding analogous processes in human, which could potentially lead to the development of novel therapeutic approaches in ROS-associated diseases treatment.

## Introduction

Reactive oxygen species (ROS) represents a group of molecules derived from oxygen, which are formed by reduction/oxidation reactions (redox reactions) or by electronic excitation. The superoxide anion radical (O_2_^.–^) and hydrogen peroxide (H_2_O_2_) are key ROS signaling agents generated by the mitochondrial electron transport chain and by more than 40 enzymes, mainly NADPH oxidases, that are regulated by growth factors and cytokines. High levels of ROS production, designated as oxidative distress or damage, result in molecular damage, leading to genome alteration and apoptotic death. At physiological and elevated levels, ROS signal via different post-translational protein modifications is referred to as ROS signaling or oxidative eustress. To date, it is well established that ROS are fundamentally important as second messenger signaling molecules in cell biology and physiology. Hydrogen peroxide is the major ROS in the redox-dependent regulation of biological processes (Thannickal and Fanburg, 2000; Reczek and Chandel, 2015). The intracellular H_2_O_2_ is maintained under a tight control in low nanomolar levels (1–100 nM). The H_2_O_2_ generation is stimulated by metabolic signals or by growth factors, chemokines, or physical stressors (Sies, 2017). At physiological nanomolar concentrations, H_2_O_2_ is the major oxidant having pleiotropic signaling functions via specific reversible oxidation of protein targets. The H_2_O_2_ regulates various signal transduction events, cell metabolism, and stress responses, being involved in numerous cellular and developmental processes (Jones and Sies, 2015). Roughly, the intracellular concentration of the superoxide anion radical is much lower than that of H_2_O_2_ (10^–11^ vs. 10^–8^ M) (Chance et al., 1979). The H_2_O_2_ removal is achieved via efficient reducing systems. Protein targets of oxidants serve as redox switches in cell signal transduction. The major mechanism of H_2_O_2_-mediated posttranslational modifications of target proteins occurs by the oxidation of sulfur groups in target proteins (Zeida et al., 2019). It can also occur via reversible methionine oxidation, protein metal center oxidation, selenoproteins, and oxidized lipids (Kaya et al., 2015; Santos et al., 2016). In addition, it has been observed that moderately elevated ROS levels regulate systemic adaptive responses to support health and longevity—the process referred to as mitochondrial hormesis or mitohormesis (Ristow and Schmeisser, 2014).

In contrast to the physiological levels, high concentrations of H_2_O_2_ (above 100 nM) cause oxidative destress by non-specific protein oxidation and damage of biomolecules, resulting in growth arrest and apoptosis. Oxidative DNA damage is involved in mutagenesis, cancer development, DNA methylation, and changes of chromatin structure (Bast and Goris, 1989; Kreuz and Fischle, 2016). The pleiotropic activities of oxidants influence a multitude of fundamental processes with extensive consequences for health and disease progression (Santolini et al., 2019).

In this review, we discuss the physiological functions of intracellular ROS, both as a redox signal and a secondary messenger of various cell signaling pathways, in the context of regulation of stem cell self-renewal and differentiation during various developmental processes in *Drosophila* and mammals. The signaling functions of ROS are ancient and conserved in various cell and developmental processes of metazoans, including regulation of stem cell populations. We accent that the functional genetics in *Drosophila* has advantages in discovering the function of ROS in fundamental developmental processes. The sources and regulation of intracellular ROS, as well as their downstream molecular targets and important functions in embryonic stem cells, nuclear reprogramming to pluripotent state, and ROS-mediated cell-non-autonomous signaling are discussed. Other aspects of ROS signaling and their biological functions have been discussed in a number of excellent reviews (Bigarella et al., 2014; Holmstrom and Finkel, 2014; Sies and Jones, 2020).

## Pathways of ROS Generation and Regulation

### Mitochondrial ROS

**FIGURE 1:**
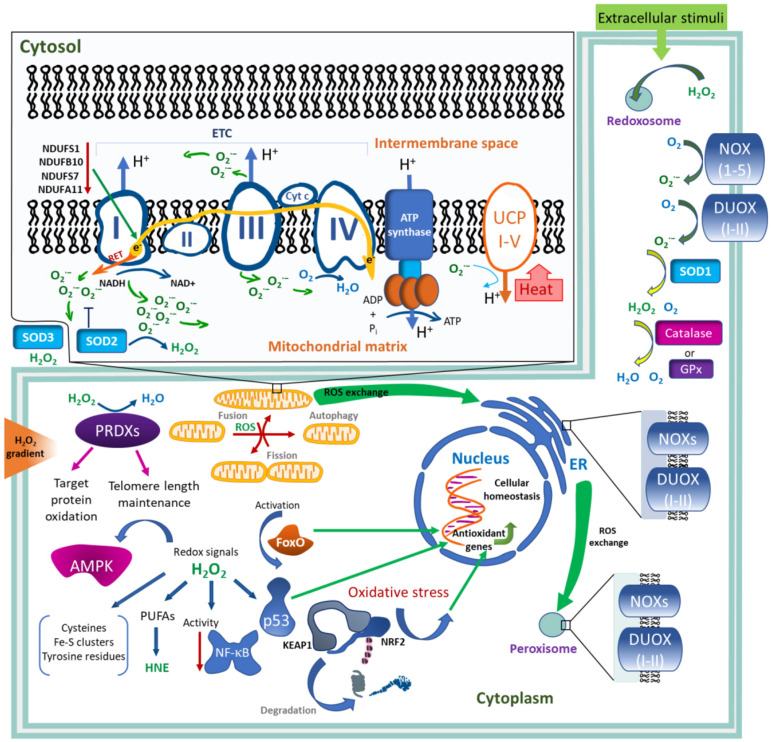
Generation and regulation of ROS levels in the cell. ROS generation by mitochondrial electron transport chain (ETC). During electron transfer through ETC complexes I, II, and III, the electrons (e-) leak and reduce molecular oxygen releasing superoxide anion (O_2_^–^) either into the matrix (CI, CII, CIII) or into the intermembrane space (CIII). Complexes I and III make the maximum contribution to the generation of O_2_^–^. Inactivation of either NDUFS1, NDUFSB10, NDUFS7, or NDUFA11 subunits of complex I leads to the overaccumulation of ROS. O_2_^–^ can be also produced by reverse electron transport (RET). Upon the generation of the ETC-mediated proton gradient, ATP synthase produces ATP, while uncoupling proteins I–V (UCP I–V) use the gradient to regulate its dissipation and heat production, which is activated by O_2_^–^. ROS play an important role in the control of mitochondrial dynamics and stability, regulating mitochondrial fission, fusion, and autophagy. In the cytoplasm, transmembrane NADPH oxidases (NOXs) and dual oxidases (DUOXI-II) are the main contributors of ROS, generating O_2_^–^ through the reduction of molecular oxygen. O_2_^–^ can be transformed into molecular oxygen and H_2_O_2_ by superoxide dismutase 1–3 (SOD2–3) in different cell compartments. Other antioxidant enzymes, catalase, peroxiredoxins (PRDXs), and glutathione peroxidases (GPx), catalyze the removal of H_2_O_2_. PRDXs contribute to telomere length maintenance. Nuclear factor erythroid derived 2/kelch-like ECH-associated protein 1 (NRF2/KEAP1), forkhead box protein O (FoxO), and p53 transcription factors sense oxidative stress and induce antioxidant gene expressions, contributing to cellular homeostasis. Redox signaling affects AMP-activated protein kinase (AMPK) and nuclear factor kappa-B (NF-κB). Peroxidation of n-6 polyunsaturated fatty acids (PUFAs) leads to the generation of biologically active aldehydes, including 4-hydroxy-2-nonenal (HNE). Redox signaling predominantly occurs through an oxidative modification of the cellular cysteines or by targeting Fe–S-clusters or tyrosine residues. Redox signals pass through many cellular compartments, such as mitochondria, nucleus, endoplasmic reticulum (ER), peroxisomes, and redoxosomes. Compartmentalization of H_2_O_2_ occurs upon extracellular stimuli, such as cytokines, growth factors, and nutrients. A 100–500-fold gradient of H_2_O_2_ exists between extracellular and intracellular spaces.

To maintain the ROS levels in cells at physiological concentrations, various mechanisms of ROS production and regulation exist, including localized generation, detoxification by antioxidant factors, and redox relays. In human, there are 41 enzymes that generate H_2_O_2_ and O_2_^–^ (Sies and Jones, 2020). The main enzymatic sources of ROS are the mitochondrial electron transport chain (ETC) (Murphy, 2009) and transmembrane NADPH oxidases (NOXs) (Bedard and Krause, 2007). H_2_O_2_ is also generated by various others, other than the NOX oxidases present in the endoplasmic reticulum (ER) and peroxisomes, as well as by several superoxide dismutases (SOD1–SOD3) mediating a localized production of H_2_O_2_ from the superoxide anion (Figure 1).

Redox regulation is among the basic physiological functions of the mitochondria, including energy generation, regulation of metabolic pathways, and epigenetic processes (Matilainen et al., 2017). Eleven different sites of electron leak resulting in superoxide anion/ROS generation have been shown in the electron transport chain (ETC) of isolated mitochondria (Brand, 2016). Based on *in vitro* experiments, it has been shown that the main sites of ROS generation are ETC complexes I (CI), II (CII), and III (CIII) (Goncalves et al., 2015). CI and CIII play a major role in ROS production *in vivo* (Sena and Chandel, 2012; Chouchani et al., 2014). Seven of these sites within the ETC include CI deposit ROS in the mitochondrial matrix, whereas CIII and glycerol 3-phosphate dehydrogenase release ROS into either the matrix or intermembrane space (Droge, 2002; Sena and Chandel, 2012; Bleier et al., 2015; Brand, 2016). The compartment-dependent ROS generation is of functional importance for the modification of redox-related proteins (Bleier et al., 2015), including several mitochondrial proteins. For example, Fe–S cluster-containing ETC components can be regulated by releasing O_2_^–^, thereby impacting mitochondrial respiration and oxidant generation. The generated ROS levels vary and depend on the status of ETC activity.

Mitochondrial complex I representing NADH:ubiquinone oxidoreductase, the largest multiunit enzyme of ETC, has an approximate size of 1 MDa and consists of 45 subunits. Its hydrophobic domain is responsible for the proton gradient potential and the hydrophilic domain is engaged in electron transfer (Zhu et al., 2016; Blaza et al., 2018). CI, localized in the mitochondrial inner membrane, oxidizes NADH generated from the tricarboxylic acid cycle to NAD^+^ by sequence redox reactions, resulting in the reduction of ubiquinone accompanied with the protons transport across the inner membrane to the intermembrane space of the mitochondria. The resulting proton gradient energy is utilized by ATP synthase, generating ATP, and transporting various low molecular and protein molecules into the mitochondrion.

CI is the main contributor to ROS generation (Figure 1). It has been proposed that the superoxide produced by electrons leak into two sites of CI. In the first site, represented by seven iron-sulfur centers, electrons transfer between the flavin mononucleotide (FMN, F-site), where NADH oxidation to NAD^+^ occurs. In the second site, represented by the coenzyme Q (CoQ) site (Q-site), electrons are finally transferred to ubiquinone (Koopman et al., 2010; Scialo et al., 2013). Normally, by supplying the ETC with CI-linked substrates (glutamate or pyruvate with malate), electrons move through CI in a forward direction (Murphy, 2009). The electron leak and superoxide formation are independent of both the redox state of CoQ and mitochondrial membrane potential. It has also been shown that ROS can be produced via the reverse electron transport (RET) mechanism. RET occurs upon supplying CII with its substrate, succinate. Under this condition, some electrons flow through CI in a reverse direction (Chance and Hollunger, 1961; Chouchani et al., 2014). Recently, it has been established that both the amount of ROS and the sites of ROS generation are important for cell signaling function (Cocheme et al., 2011). Based on this, it has been proposed that the regulation of ROS generation at specific sites of mitochondrial ETC can induce various specific physiological signaling events, and that site-specific antioxidants can be potentially used in therapies (Orr et al., 2015; Brand et al., 2016; Scialo et al., 2017).

Mitochondrial fusion and fission, as well as cristae remodeling, regulate the morphology of the mitochondrial network and functions. These processes are also in a tight bidirectional relationship with oxidants. The state of the mitochondrial network relies on respiration and ROS production. In contrast, oxidants regulate the functions of the proteins involved in mitochondrial network dynamics (Murley and Nunnari, 2016; Bulthuis et al., 2019). Peroxisome proliferator-activated receptor-γ co-activator 1α (PGC1α) is activated by ROS and regulates mitochondrial biogenesis, connecting redox signaling to the mitochondrial network regulation. ROS also regulates mitochondrial quality via autophagy by removing dysfunctional mitochondria. In this case, low ROS levels trigger the selective removal of mitochondria in a fission-dependent mitophagy, whereas higher oxidant levels lead to non-selective macroautophagy (Frank et al., 2012). Mitochondrial uncoupling results in decoupling the ETC work from ATP synthesis, resulting in a proton leak. This leads to a dissipation of the electrochemical energy as heat, presenting a key mechanism of thermogenesis. The regulation of this process is mainly mediated by uncoupling proteins 1–5 (UCP1–5) (Berry et al., 2018). Mild mitochondrial uncoupling is mediated by the activation of the uncoupling function of UCP by a superoxide anion (Echtay et al., 2002). This causes a limitation in the activity of the ETC, providing a negative feedback in the ROS production by the mitochondria.

#### Drosophila Studies of ETC Complexes

Mitochondria is an ancient cell organelle and its genetics and function are very conserved among metazoan. *Drosophila* is an excellent genetic model to study the mitochondrion electron transport chain biogenesis and function, as well as the different physiological and pathological processes induced by mitochondrial ROS. Having several important functions, ETC CI is also a major source of reactive oxygen species (Owusu-Ansah et al., 2008; Sanz et al., 2010; Rhooms et al., 2020). Inactivation of the core NDUFS1 (fly homolog–ND75) or accessory NDUFB10 (fly homolog–ND-PDSW) subunits of CI in different *Drosophila* tissues, including epithelia and blood, causes an increased ROS production (Owusu-Ansah et al., 2008; Owusu-Ansah and Banerjee, 2009; Sinenko et al., 2012). The consequences of ROS generation in various fly tissues will be discussed in the next section. Loss-of-function mutations in the NDUFS7 or NDUFA11 subunits of *C. elegans* also result in an increased ROS (Rauthan et al., 2015).^1^ *Drosophila* flight muscles CI biogenesis proceeds via the formation of ∼315, ∼550, and ∼815 kDa complex assembly intermediates. It was defined that dNDUFS5 is required for converting ∼700 kDa into the ∼815 kDa CI assembly intermediate (Garcia et al., 2017).

### Cytosol ROS and Their Regulation in the Cell

It has been shown that the contributions by NOXs and ETC to ROS generation are close to equal, while it depends on the contexts and cell type (Wong et al., 2019). In cytosol, NADPH oxidases NOX (1–5), including dual oxidases DUOX (1–2), and other oxidases generate O_2_^–^/H_2_O_2_ in various subcellular compartments, including the endoplasmic reticulum (ER) and peroxisomes. Extracellular stimuli, such as cytokines, growth factors, and nutrients, promote the compartmentalization of H_2_O_2_ in specialized redox-active endosomes (redoxosomes) involved in local regulation or cell signaling (Mishina et al., 2013; Spencer and Engelhardt, 2014).

ROS are maintained at steady-state levels via a tight control of their sources and scavenging (Figure 1). Levels of H_2_O_2_ are regulated by various scavenging or antioxidant enzymes. Catalase dismutates H_2_O_2_ to H_2_O and O_2_ or it reduces H_2_O_2_ to H_2_O by oxidizing hydrogen-donating compounds (Chance et al., 1979). Peroxiredoxins (PRDXs) and glutathione peroxidases (GPx) catalyze the removal of H_2_O_2_, contributing to the regulation of its levels in the cell (Figure 1; Rhee and Kil, 2017; Brigelius-Flohe and Flohe, 2019). Nicotinamide nucleotide transhydrogenase (NNT) also regulate H_2_O_2_ levels by supporting the thioredoxin and the glutathione antioxidant system (Hanschmann et al., 2013). There is a 100–500-fold gradient of H_2_O_2_ concentration between the extracellular and intracellular spaces (Lyublinskaya et al., 2018; Mailloux, 2018; Pak et al., 2020; Sies and Jones, 2020). Control of intracellular H_2_O_2_ gradients is maintained mainly by the thioredoxin system and by exchanges between the different cell compartments including the ER, mitochondria, and peroxisomes (Appenzeller-Herzog et al., 2016; Yoboue et al., 2018; Fransen and Lismont, 2019; Mishina et al., 2019). In neutrophils, myeloperoxidase functions to generate hypochlorous acid from H_2_O_2_, acting as a defense oxidant against pathogens (Winterbourn et al., 2016). In general, short-lived ROS are mainly involved in intracellular signaling, albeit they can also participate in intercellular communications (Mishina et al., 2013).

### Antioxidant Response Systems and Targets of Redox Signaling

The system based on nuclear erythroid-related factor 2 (NRF2) and kelch-like ECH-associated protein 1 (KEAP1) is a major sensor of oxidative stresses, regulating redox homeostasis in eukaryotes. KEAP1 harbors several cysteine residues that can be oxidized, sensing the oxidative stress, and functioning as an NRF2 inhibitor (Yamamoto et al., 2018). A conformational change of KEAP1 upon oxidation inhibits the ubiquitylation of transcription factor NRF2, allowing its translocation to the nucleus to activate the expression of antioxidant genes (Yamamoto et al., 2018). The NRF2–KEAP1 system can be regulated by thioredoxin reductase 1 and by the sirtuin family of deacetylases (Figure 1; Cebula et al., 2015; Singh et al., 2018).

The forkhead box protein O (FoxO) family of transcription factors contributes to the maintenance of cellular homeostasis by integrating the redox signals from other signaling pathways, thereby regulating a wide spectrum of genes including antioxidants. This occurs via direct cysteine oxidation in FoxO members as well as via oxidant-mediated upstream regulatory mechanisms (Eijkelenboom and Burgering, 2013; Klotz and Steinbrenner, 2017). Transcription factor p53 is also regulated by oxidants. H_2_O_2_ modulates the selective transactivation of p53 target genes, probably by the direct oxidation of p53 cysteine residues and indirect modulation of signaling networks. In turn, p53 regulates the expression of antioxidant genes that control the cellular redox balance (Liu et al., 2008). Nutrient or energy stress and excess or deprivation of nutrients are also associated with redox signaling and increased oxidant formation. These include pro-growth and -proliferation signaling of AMP-activated protein kinase (AMPK) and mechanistic target of rapamycin kinase (mTOR) which sense glucose and amino acid availability, correspondingly. Regulation of energy stress, mediated by AMPK, is under redox control. AMPK is not activated by its own oxidation but is indirectly affected through (1) decreased mitochondrial respiration and (2) amounts of generated energy (Hinchy et al., 2018).

Hypoxia, i.e., low concentrations of oxygen (below 5% O_2_), is sensed and responded to in cell by a transcription factor, hypoxia-inducible factor (HIF) (Kaelin and Ratcliffe, 2008). Hypoxia leads to an increase of ROS generation as it inhibits mitochondrial ETC activity (Hernansanz-Agustin et al., 2017). In addition, mounted ROS levels stabilize HIF during hypoxia, additionally activating a hypoxic response (Chandel et al., 2000; Waypa et al., 2016). HIF prolyl hydroxylases are modulated by oxidants, which sense oxygen availability and drive HIF hydroxylation and subsequent proteasomal degradation. Oxidants also affect HIF pathways in normoxic conditions, involving in a stress-adaptive response (Pouyssegur and Mechta-Grigoriou, 2006). The transcription factor nuclear factor-κB (NF-κB) functions as a master regulator of inflammation. Inflammation is associated with extensive H_2_O_2_ production which, depending on the context, have both stimulatory and inhibitory roles in NF-κB function. H_2_O_2_ via oxidation and activation of the inhibitor of NF-κB (IκB) kinases can regulate the NF-κB pathway (Oliveira-Marques et al., 2009). It can also directly modulate the DNA-binding activity of NF-κB. As such, nuclear H_2_O_2_ reduces NF-κB transcriptional activity, while H_2_O_2_ scavenging by peroxidoxin 1 (Pxn1) stimulate the activity (Halvey et al., 2007).

Peroxiredoxins (PRDXs) can oxidize target proteins, for instance, PRDX2 modifies the transcription factor STAT3 and MAPK kinase (MEKK4, and its fly homolog MAP3K) in the p38 signaling pathway (Barata and Dick, 2020; Sobotta et al., 2015). Peroxiredoxin 1 (PRDX1) is also involved in telomere length maintenance by counteracting the H_2_O_2_-mediated oxidative damage of telomeric DNA and promoting telomere elongation (Ahmed and Lingner, 2018). Redox signaling predominantly occurs through oxidative modification of the cellular cysteines (represent 10–20% of the all thiols). Fe–S-clusters or tyrosine residues are also oxidant targets that have potential signaling activities. Protein-tyrosine phosphorylation is impacted by a direct redox-based regulation of protein tyrosine phosphatases and protein-tyrosine kinases (Kamata et al., 2002; Truong et al., 2016; Dustin et al., 2020; Londhe et al., 2020). Thus, oxidants exert modulatory effects on transcriptional regulation, DNA damage response, DNA repair, cell cycle, and DNA replication, meanwhile redox signaling affects protein function including enzyme activity, heat shock protein, signal transduction, and chromatin factors functions (Brigelius-Flohe and Flohe, 2011; Bak et al., 2019; Young et al., 2019).

Peroxidation of membrane phospholipids, such as linoleic acid and arachidonic n-6 polyunsaturated fatty acids, by ROS leads to a production of reactive aldehydes: malondialdehyde (MDA) and, in particular, 4-hydroxy-2-nonenal (HNE). HNE is the major and most extensively studied aldehyde which easily reacts with a wide range of proteins and low-molecular-weight compounds, such as glutathione. To date, multiple studies showed the various biological activities of HNE in different cells of metazoans (Parola et al., 1999; Moneypenny and Gallagher, 2005). High concentrations of HNE are detrimental for cells, causing irreversible damage of cell functions and leading to growth arrest and cell death. However, low to moderate HNE concentrations (up to 1 μM) display signaling activities in various types of cells, including the regulation of NF-kB, Nrf2, AKT, and mTOR signaling molecules (Dianzani, 2003; Barrera et al., 2008; Zhang and Forman, 2017). It has been shown that the second messenger of HNE induces mitochondria uncoupling through UCP1–3 proteins and adenine nucleotide translocase (ANT) and, thus, decreases mitochondrial ROS production (Echtay et al., 2003).

## Physiological ROS Signaling

### ROS Function in Adult Stem and Progenitor Cells

**FIGURE 2:**
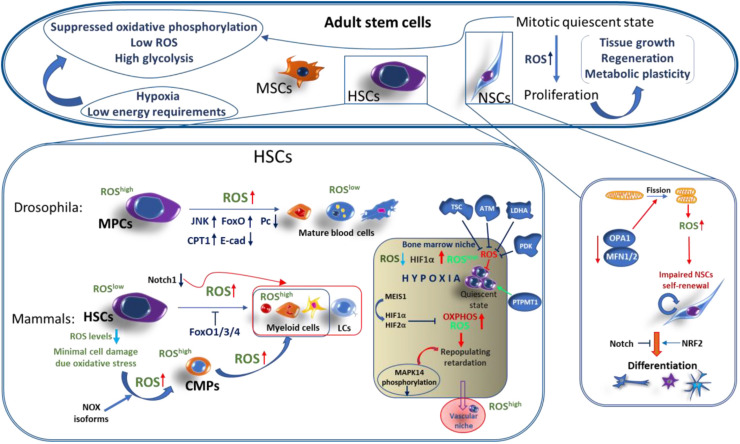
ROS functions in adult stem cells. Adult stem cells (ASCs), such as hematopoietic stem cells (HSCs), neural stem cells (NSCs), and mesenchymal stem cells (MSCs), are maintained under hypoxic conditions in a mitotically quiescent state and are featured by low ROS levels, suppressed oxidative phosphorylation, high glycolysis rate, and low energy requirements. Increased levels of ROS is an important factor in mediating the proliferation and differentiation of ASCs. In mammals: HSCs are maintained in a quiescent state in hematopoietic bone morrow and vascular niches. HSCs in the hematopoietic bone morrow niche expressing minimal levels of ROS are characterized by increased stemness capacities over HSCs in vascular niche which has a higher ROS levels. Several factors providing the maintenance of low HSC ROS levels within the hypoxic bone morrow niche and controlling their mitotic quiescence and stemness include: Notch1, MEIS1-controll hypoxia-inducible factor 1 and 2 alpha (HIF1α/ HIF2α), tuberous sclerosis complex (TSC), ataxia telangiectasia mutated (ATM) protein, lactate dehydrogenase A (LDHA), PTEN-like mitochondrial phosphatase (PTPMT1), and pyruvate dehydrogenase kinase (PDK). Upon differentiation, HSCs-derived common myeloid progenitors (CMPs) start the expression of high levels of ROS, mediated by NOX enzymes and triggered by the downregulation of FoxO1/3/4 transcription factors. Further CMPs differentiate into myeloid lineage cells associated with a further gradual increase of ROS generation. In *Drosophila*: Myeloid-type progenitor cells (MPCs), similar to mammalian CMPs, are maintained under a tight regulation of relatively high ROS levels, while differentiated blood cells are featured by a low ROS production. Importantly, the increase of ROS upon oxidative eustress causes the robust differentiation of MPCs into all types of mature blood cells, which is accompanied by the activation of the JNK/FoxO and JNK/carnitine palmitoyltransferase 1(CPT1) axes, downregulation of polycomb (Pc) chromatin remodeling protein, and reduction of E-cadherin expression. In mammalian NSCs: Mitochondrial dynamics through modifying ROS signaling regulates NSCs fate. Dynamin-like protein (OPA1) or mitofusins-1/2 (MFN1/2) mediate mitochondrial fusion. Inactivation of these proteins leads to mitochondrial fragmentation or fission, resulting in an increased ROS production which, in turn, suppresses the NSCs self-renewal through the downregulation of Notch1 and activation of NRF2 pathways, triggering NSC differentiation.

Adult stem cells contribute to the replenishing of aged or damaged tissue during the life of a metazoan. These can be multipotent or specialized stem cells that are limited to differentiate to specific tissue types and reside in specialized stem cell niches. In contrast to embryonic stem cells (see below), adult stem cells self-renew and are maintained mainly in a mitotic quiescent state, being able to quickly proliferate during tissue growth or in response to tissue damage (Wilson and Trumpp, 2006; Bigarella et al., 2014). These processes are tightly regulated at various levels, including metabolic plasticity, preventing the exhaustion of these cells. For instance, maintenance of hematopoietic stem cells (HSCs) is dependent on glycolysis while a switch to mitochondrial respiration occurs in downstream progenitors and upon differentiation, reviewed elsewhere (Warr and Passegue, 2013; Ito and Suda, 2014; Folmes and Terzic, 2016). Importantly, different types of ASC, including HSCs, neural stem cells (NSCs), and mesenchymal stem cells (MSCs), have low levels of ROS and are enriched by glycolytic metabolites (Tothova et al., 2007; Simsek et al., 2010; Suda et al., 2011; Yeo et al., 2013). The suppressed oxidative phosphorylation in ASCs is linked to their hypoxic niche location, low energy requirements, and minimization of mitochondrial ROS-mediated oxidative stress (Figure 2; Jang and Sharkis, 2007; Eliasson and Jonsson, 2010; Kunisaki et al., 2013; Urao and Ushio-Fukai, 2013).

#### In *Drosophila*

**FIGURE 3:**
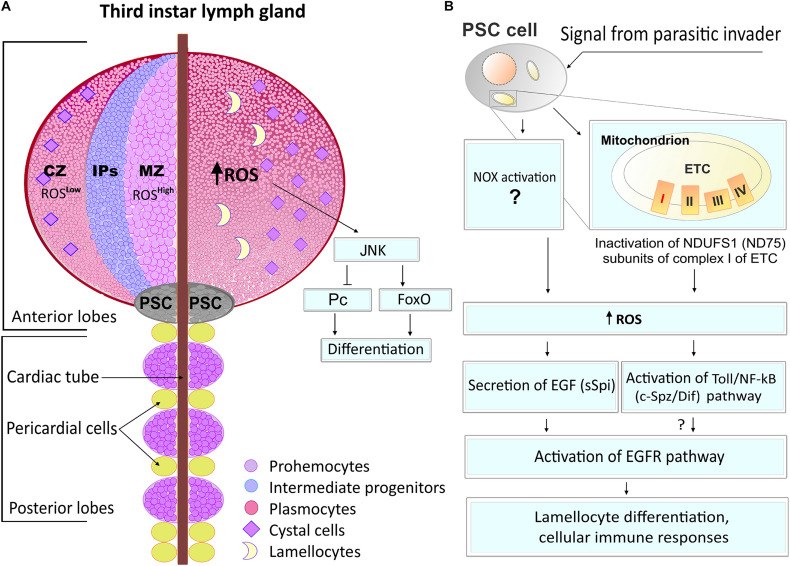
ROS functions in *Drosophila* hematopoiesis. **(A)** The *Drosophila* hematopoietic organ, lymph gland, consists of anterior and posterior lobes (left panel). In the anterior lobe, there are three zones of cell differentiation: cortical zone (CZ), intermediate progenitor zone (IPs), and medullary zone (MZ). Prohemocytes of MZ are featured by high levels of ROS while differentiated cells (plasmacytes and crystal cells) have much less ROS levels. An increase of the ROS levels in prohemocytes leads to JNK pathway activation which, through FoxO and Pc, promotes cell differentiation. **(B)** Hematopoietic niche, also called posterior signaling center (PSC), is located in the posterior side of the anterior lobe of the lymph gland. PSC cells can sense oxidative stress and regulate the cellular immune response in larva hematopoietic organ and in hemolymph. The parasitic wasp pathogen infection or inactivation of the NDUFS1(fly ND75) subunit of complex 1 ETC induces ROS generation in the niche cells, resulting in the secretion of an epidermal growth factor (EGF, fly sSpitz, sSpi) cytokine signal. This leads to an activation of the epidermal growth factor receptor (EGFR) pathway and the lamellocyte differentiation, both involved in cellular immune responses. In parallel, ROS increase in PSC also activates the Toll/NF-κB (fly Spatzle/Dif, c-Spz/Dif) pathway, leading to cellular immune responses, presumably via EGFR pathway activation.

It was originally shown that a disruption of complex I of the mitochondrial ETC specifically retards the cell cycle during the G1-S transition by the ROS-induced JNK/FoxO/p27 signaling. This was first identified in *Drosophila* undifferentiated epithelial cells, establishing that mitochondria and, particularly, ETC CI can engage sublethal levels of ROS as specific signaling molecules to modulate cell cycle progression (Owusu-Ansah et al., 2008). Furthermore, the authors demonstrated the important signaling function for ROS in the *Drosophila* hematopoietic system (Owusu-Ansah and Banerjee, 2009). It was shown that multipotent hematopoietic myeloid-type progenitor cells maintained the increased levels of ROS under physiological conditions. Downregulation of ROS occurs in differentiated cells, while scavenging the ROS from hematopoietic progenitors suppresses their differentiation. In contrast, the increase of ROS above the basal level leads to a precocious differentiation of the hematopoietic progenitors into mature blood cell types via the activation of the JNK and FoxO signaling and downregulation of the Polycomb (Pc) transcriptional repressor (Figure 3A). These data suggest that the physiological (moderately high) ROS level in the progenitor population regulates their maintenance, while ROS concentrations below these levels play a signaling role in the regulation of hematopoietic cell fate (Owusu-Ansah and Banerjee, 2009). It was shown that ROS control E-cadherin expression (Gao et al., 2014) as well as its signals through JNK, activating fatty acid β-oxidation (Tiwari et al., 2020) to promote the differentiation of hematopoietic progenitors by reducing E-cadherin level and inducing histone modification, correspondingly.

#### In Mammals

A similar ROS function was observed in the mammalian hematopoietic development. Tothova et al. (2007) showed that FoxO knockout mice displayed increased ROS in hemopoietic stem cells resulting in a loss of their proper self-renewal and viability, causing myeloid differentiation. ROS levels are tightly regulated in HSCs and early myeloid progenitors, and this regulation plays an important role during hematopoiesis and myelopoiesis, and it also protects these cells from damage caused by oxidative stress. In hematopoietic stem cells, ROS production is minimal while the common myeloid progenitors (CMPs) produce significantly increased levels of ROS required for their maintenance (Figure 2). The NADPH oxidases play a key role in intracellular ROS generation in human HSCs (Piccoli et al., 2007). Different NOX isoforms may have a functional importance at varying stages of myeloid cell differentiation and in ROS generation by mature myeloid cells during innate immune responses.

The importance of a low ROS level for maintaining quiescence, self-renewal, and long-term survival of multipotent HSCs has been shown. Several pathways regulating the ROS levels are involved in these processes. HSCs are maintained in a quiescent state under a hypoxic environment of osteoblastic niches within the bone marrow (Eliasson and Jonsson, 2010; Eliasson et al., 2010). The quiescence of HSCs is regulated by an increased expression of hypoxia inducible factor 1 alpha (HIF1α). HIF1α/HIF2α deficiency has retarded the long-term repopulating function in human HSCs via activated oxidative phosphorylation and increased ROS production (Takubo et al., 2010; Rouault-Pierre et al., 2013). Homeobox protein MEIS1 regulates both HIF1α and HIF2α, acting as an important regulator of metabolism and ROS signaling upstream of HIF in HSCs (Figure 2; Simsek et al., 2010; Kocabas et al., 2012).

Increased ROS and phosphorylation of p38 mitogen-activated protein kinase, p38-MAPK (MAPK14), in FoxO3a-deficient HSCs also leads to a defective quiescence (Miyamoto et al., 2007). It was also shown that among defined ROS^low^ and ROS^high^ populations in mice, the ROS^low^ HSC population residing in the osteoblastic niche displayed greater self-renewal and repopulating capacities associated with a reduced phosphorylation of p38-MAPK (Jang and Sharkis, 2007). Whereas, the ROS^high^ cells residing in the vascular niche, closer to peripheral blood, undergo differentiation. Oxidative stress induced in HSCs of ataxia telangiectasia-mutated (ATM) knockout mice leads to a defect in the HSC function, resulting in a progressive bone marrow failure (Ito et al., 2004). It was reported that the tuberous sclerosis complex (TSC) (a component target of the rapamycin, mTOR, pathway) deletion resulted in increased ROS levels in HSCs, causing the lack of quiescence, affecting their self-renewal, and reducing hematopoiesis (Chen et al., 2008). It has also been shown that elevated ROS levels originating from the mitochondria induce murine HSCs differentiation that is mediated by NOTCH1 suppression (Cao et al., 2016). ROS also regulate the differentiation of HSCs to terminally differentiated myeloid progenitors, as well as megakaryocyte differentiation into mature platelets (Figure 2; Motohashi et al., 2010).

Conditional deletion of lactate dehydrogenase A (LDHA) induces an increase in ROS, causing blood stem and progenitor cell defects (Wang et al., 2014). HSCs leave the quiescence state and start to proliferate upon shifting from glycolysis to oxidative phosphorylation. Pyruvate dehydrogenase kinase (PDK) and PTEN-like mitochondrial phosphatase (PTPMT1) are the key regulators of pyruvate oxidation by the mitochondria. It has been shown that a genetic ablation of PDK in mice increases oxidative phosphorylation and ROS generation, leading to a loss of quiescence and exhaustion of the HSC pool (Takubo et al., 2013). As opposed to that, enhancing glycolysis by PTPMT1 inactivation in mice resulted in a robust expansion of the HSC population and prevented their differentiation (Yu et al., 2013). Physiological ROS-mediated signals induced by changes in mitochondrial dynamics are important to regulate neuronal stem cell self-renewal and differentiation. These ROS signals induced by the lack of mitochondrial fusion due to the deletion of dynamin-like protein (OPA1) or mitofusins-1/2 (MFN1/2) lead to an impaired NSC self-renewal. These signals trigger a dual program to suppress self-renewal and to promote differentiation of NSC via ROS and NRF2-mediated retrograde signaling (Figure 2; Khacho et al., 2016).

### Role of ROS in Various Stem Cells in *Drosophila*

**FIGURE 4:**
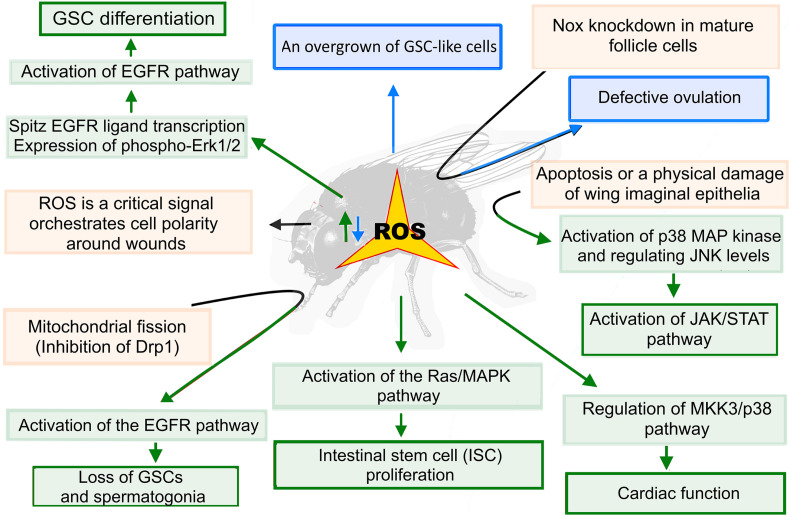
ROS functions in *Drosophila* stem cells and tissue regeneration. Redox homeostasis plays important roles in the maintenance of stem cells in *Drosophila*. High ROS levels (highlighted in green) induce germline stem cells (GSC) differentiation via the activation of the EGF/EGFR/Erk1/2 pathway. On the other hand, reduced ROS levels (highlighted in blue) promote the overgrowth of GSC-like cells. The accumulation of ROS caused by mitochondrial fission (inhibition of Drp1) leads to an activation of the EGFR pathway in adjacent somatic cyst cells in *Drosophila* larval testes leading to the loss of GSCs and spermatogonia. The ROS act in a paracrine fashion to regulate normal cardiac function. In pericardial cells (PCs), ROS stimulate the downstream MKK3/p38 MAPK pathway that regulates the cardiac function of the cardiomyocytes (CMs). Apoptosis or a physical damage of the wing imaginal epithelia also leads to a ROS-induced activation of the p38 MAP kinase, which induces moderate levels of JNK-mediated Upd cytokine expression, triggering the JAK/STAT pathway activation and cell regeneration processes.

It has been shown that redox homeostasis regulated by Keap1/Nrf2 signaling plays important roles in the maintenance of germline stem cells (GSCs) in *Drosophila*. Such high levels of ROS lead to a decrease in GSC number by promoting their precocious differentiation. In contrast, low ROS levels promoted the growth of GSC-like cells. Authors showed that high ROS levels induce GSC differentiation via the activation of EGFR signaling by enhancing the spitz EGFR ligand transcription and expression of phospho-Erk1/2 (Figure 4; Tan et al., 2017). A similar mechanism was identified in the regulation of the systemic cellular immune response by the hematopoietic niche (Sinenko et al., 2012). It was also shown that the accumulation of ROS caused by mitochondrial fission (inhibition of Drp1) in early germ cells resulted in the loss of germline stem cells and spermatogonia due to the activation of the EGFR pathway in adjacent somatic cyst cells of *Drosophila* larval testes (Senos Demarco and Jones, 2019). Recently, a novel histone acetyltransferase Kat8 regulating antioxidant gene expression was identified to be essential for oogenesis and female fertility (Yin et al., 2017). Altering the activity of ROS scavenger, SOD1, revealed that ROS levels are critical in maintaining the migration of embryonic primordial germ cells (PGCs) (Syal et al., 2020). NOX cytoplasmic superoxide-generating enzyme is involved in the regulation of *Drosophila* ovulation. *Nox* knockdown in mature follicle cells leads to a defective ovulation mediated via ROS reduction. NOX enzymatic activity is controlled by the Octopamine/OAMB-Ca^2+^ signaling pathway. The authors showed that extracellular SOD3 acts as a key signaling molecule for follicle rupture, and they suggested that the same mechanism may exist in regulating ovulation in mammals (Li et al., 2018).

High ROS levels and oxidative stress have been shown to be necessary to induce intestinal stem cell (ISC) proliferation in *Drosophila*. It was identified that regulators of cytosolic Ca^2+^ levels, transient receptor potential A1 (TRPA1) and ryanodine receptor, were required for the oxidative stress-induced activation of the Ras/MAPK pathway, which, in turn, drove ISC proliferation (Xu et al., 2017). A role of ROS in mediating interactions between pericardial cells (PCs) and neighboring cardiomyocytes (CMs), which is important for the heart function in *Drosophila*, has also been shown. Under normal conditions, PCs contain elevated levels of ROS compared to the neighboring CMs. The ROS act in a paracrine fashion to regulate a normal cardiac function (Figure 4). In PCs, ROS elicit downstream MKK3/p38 MAPK signaling pathway, cell-non-autonomously regulating cardiac function of CMs (Lim et al., 2014).

It was identified in *Drosophila* and zebrafish embryos that ROS was a critical signal orchestrating cell polarity around wounds. In *Drosophila*, ROS drive wound healing in part through an ortholog of the Src kinase, Src42A, which acts as a redox sensor promoting the polarization of junctions and the cytoskeleton around wounds. It was proposed that ROS act as a reparative signal that drives rapid embryonic wound healing in vertebrate and invertebrate species (Hunter et al., 2018). During tissue repair and regeneration, apoptotic stimuli in epithelial cells induce proliferation in neighboring cells to compensate the overall cell loss. This process is called apoptosis-induced compensatory proliferation (AIP). Along with its proapoptotic function, effector caspases are also directly involved in the generation of the signals required for AIP. It was shown that apoptotic caspases mediated the ROS generation for promoting AIP (Diwanji and Bergmann, 2018). Santabarbara-Ruiz and colleagues (2015) demonstrated that a programmed cell death or a physical damage in the wing imaginal discs generates a burst of ROS, inducing the propagation of neighboring cells survival. Infiltrated ROS activate p38 MAP kinase and induce moderate levels of Jun N-terminal kinase (JNK), inducing the expression of Upd cytokine (Figure 4). In turn, the latter triggers the JAK/STAT signaling pathway required for regeneration (Santabarbara-Ruiz et al., 2015). In other studies, it has also been shown that upon damage of imaginal disc epithelial cells, a robust generation of ROS occurs in these cells, leading to an activation of the JNK and p38 signaling pathways, as well as to the stimulation of regenerative growth (Serras, 2016). The described ROS functions in various fly stem and undifferentiated cells above indicate the possible functions of redox signaling in the regulation of the maintenance and differentiation of their mammalian counterparts; however, these functions remain to be determined.

### ROS Function in Hematopoietic Niche Cells in *Drosophila*

In the *Drosophila* hematopoietic organ, the lymph gland, there are specialized hematopoietic niche cells that orchestrate the maintenance and differentiation of hematopoietic progenitors (Jung et al., 2005; Koch and Radtke, 2007; Sinenko et al., 2009). It has been shown that the hematopoietic niche cells can sense oxidative stress and regulate the cellular immune response in the larva hematopoietic organ and hemolymph. Our studies established that parasitic wasp pathogen infection induces ROS in the niche cells, resulting in the secretion of the epidermal growth factor (EGF) cytokine signal. This signal, via the activation of the epidermal growth factor receptor (EGFR) pathway, leads to the differentiation of specialized cells, lamellocytes, involved in innate immune responses (Figure 3B; Sinenko et al., 2012). This study established that the inactivation of the NDUFS1 subunit of complex 1 ETC in the niche cells caused an overproduction of ROS that mediated the parasite pathogen-induced cellular immune response. Other studies showed that during this process, ROS activate the Toll/NF-κB pathway in the PSC in response of *Drosophila* to a wasp pathogen. These EGFR and Toll/NF-κB signaling pathways act in parallel, and their simultaneous activation by elevated ROS levels in the niche cells promotes hematopoiesis and cellular immune response (Figure 3B; Louradour et al., 2017).

The cellular immune response to parasites in *Drosophila* can be compared to an emergency hematopoiesis in mammals. In the latter organisms, systemic microbial infection induces an emergency granulopoiesis characterized by a *de novo* production of neutrophils in the bone marrow (Zhao and Baltimore, 2015). The Toll-like receptor (TLR)/NF-kB pathway participated in this process in the endothelial cells of a vascular niche component (Boettcher et al., 2014). However, to date, the role of ROS in this particular process and in the EGFR pathway is largely unknown. In mice, EGFR is expressed in bone marrow HSCs, and EGF administration promotes the recovery of the HSC pool and mouse survival after a lethal dose of irradiation (Doan et al., 2013). Taken together, it will be important to determine whether the regulatory network in which ROS dually regulate the EGFR and Toll/NF-kB signaling pathways operates in mammalian innate immune cells during a pathogen-induced hematopoietic recovery upon stress.

### Pathological ROS Signaling in Hematopoietic Malignances

#### In *Drosophila*

*Drosophila* genetic model has been extensively used for the investigation of human oncogenes and tumor suppressors in terms of identifying the novel genes and components that are involved in the particular oncogenic processes (Sinenko et al., 2002, 2010; Asha et al., 2003; Sinenko and Mathey-Prevot, 2004; Evans et al., 2007; Polesello et al., 2011). In this regard, one of the leukemogenic proteins, chimeric acute myeloid leukemia-1/ETO (AML1-ETO) protein, has been investigated by *in vivo* genetic screens to identify the genes involved in the oncogenic pathway. It has been shown that AML1-ETO-induced hematopoietic progenitors expressed high levels of ROS, and SOD2- and catalase-mediated scavenging of these ROS leads to a complete suppression of the malignant phenotype (Sinenko et al., 2010). In addition, we have identified steroid hormone nuclear receptor, ecdysone receptor B1 (EcRB1), and forkhead box O (FoxO) transcription factor as the factors regulating the ROS level in leukemogenic cells. It was also found that ROS is involved in the regulation of leukemogenic transformation in *Drosophila* caused by an overexpression of the mutant forms of isocitrate dehydrogenase (IDH, IDH-R195H) (Reitman et al., 2015). These results suggest an important signaling function of ROS in the maintenance and proliferation of oncogene-transformed blood progenitor cells in flies and point to an analogous oncogenic function in mammals.

#### In Mammals

To date, it has become well documented that ROS levels are elevated and play a growth promoting function in cancer cells (Tong et al., 2015). There is an evidence of increased ROS levels in various myeloid neoplasms: in myelodysplastic syndrome and myeloproliferative neoplasms (Neukirchen et al., 2012; Marty et al., 2013; Ivars et al., 2017)and chronic myelomonocytic and myeloid leukemia (Hole et al., 2011; Aurelius et al., 2017). ROS drives leukemogenesis in acute myeloid leukemia (AML). Redox dysregulation caused by ROS promotes proliferation, differentiation, genomic, and epigenetic alterations; immune evasion; and survival in leukemic cells. ROS act as signaling molecules to regulate redox-sensitive transcriptional factors, enzymes, oncogenes, and other downstream effectors (Hole et al., 2013; Zhou et al., 2013).

An increase (in some samples, up to 100-fold) in superoxide production was observed in 65% of primary AML blasts compared with normal myeloblasts (Hole et al., 2011, 2013). The role of mutant receptor kinases in driving ROS production in AML has been shown. ROS elevation caused by activated Ras-related C3 botulinum toxin substrate (Ras) induces growth factor independent proliferation, remaining an intact antioxidant expression in leukemic blasts (Hole et al., 2011; Yang X. et al., 2013). Increased levels of ROS also drive a K-Ras myeloproliferative disease development in the murine model. The ROS in AML is provided by members of the NOX oxidases. The NOX-derived ROS production is enhanced by mutations in the FMS-like tyrosine kinase 3 (FLT3) and Ras (Sillar et al., 2019). Constitutive activation of NOX2 oxidase, resulting in ROS overproduction, occurs in more than 60% of patients with AML. Recently, it has been shown that ROS stimulate the expression of uncoupling protein 2 (UCP2) protein and phosphorylation of AMPK, promoting the expression of a key regulatory glycolytic enzyme, 6-phosphofructo-2-kinase/fructose-2,6-bisphosphatase (PFKFB3). The induction of the positive glycolytic regulator PFKFB3 by high ROS levels promotes the proliferation of leukemic cells. The studies identified PFKFB3 as a novel therapeutic target in AML (Robinson et al., 2020).

### ROS Function in Embryonic Stem Cells

**FIGURE 5:**
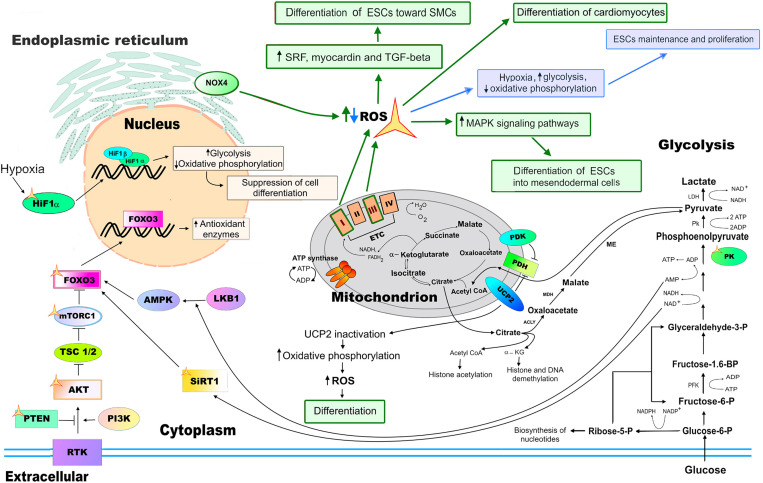
ROS functions in pluripotent stem cells (PSCs). The balance between self-renewal and differentiation of PSCs is partly regulated by ROS which are mediators of cellular redox state. Increased levels of ROS (highlighted in green) caused by prolonged hypoxia, activation of oxidative phosphorylation by inactivation of the uncoupling protein 2 (UCP2), and mitochondrial dysfunction (disruption of the functioning of complexes I and/or III of the ETC of mitochondria) all lead to reduced self-renewal control and increased ESCs differentiation. Nox4-generated ROS are involved in the proper differentiation of mouse ESCs into smooth muscle cell (SMC) lineage. Decreased ROS levels (highlighted in blue), which enhances glycolysis, or suppressing oxidative phosphorylation improve ESC self-renewal, while repressing their differentiation. SIRT1, as well as the serine–threonine liver kinase B1 (LKB1)/AMP-activated protein kinase (AMPK) pathway, activate FoxO3 transcription factor, upregulating antioxidant enzyme expression in ESCs upon oxidative stress and metabolic changes, respectively. Pyruvate dehydrogenase kinase (PDK) and UCP2 negatively regulates the pyruvate dehydrogenase (PDH) complex, controlling the level of Acetyl-CoA and further protein acetylation. The activity of the components of the receptor tyrosine kinase (RTK) PI3K/AKT/mTOR pathway, the phosphoinositide 3-kinase (PI3K), RAC-alpha serine/threonine-protein kinase (AKT), mammalian target of rapamycin (mTOR), PTEN, FoxO, as well as hypoxia inducible factor-a (HIF1a), SIRT1, and pyruvate kinase (PK) are all regulated by ROS (marked with a yellow asterisk).

Embryonic stem cells (ESCs) originate from the inner cell mass of the mammalian blastocyst and possess the ability to self-renew and differentiate into all cell types of an adult organism. All pluripotent stem cells (PSCs), including ESCs, have an enormous potential of applications in cell therapy and modeling human diseases, thus making investigation on the molecular network regulating their maintenance, including metabolism pathways and redox signaling, of great importance (Murry and Keller, 2008; Yamanaka, 2020; Sinenko et al., 2021). ESCs and other PSCs have a high proliferation rate due to a shortened G1 cell cycle phase, which requires a high supply for anabolic precursors necessary for genome and cell replication. Also, these cells have a high demand for ATP, which is the main energy source. Under the condition of a reduced oxygen supply, PSCs require a high supply during anabolic and catabolic processes. During early embryogenesis, PSCs adapt their metabolism, which includes the specific switch to glycolysis and the pentose phosphate pathway, as well as the suppression of oxidative phosphorylation with changes in mitochondrial morphology and function, membrane potentials, and ETC compositions (Prigione et al., 2010; Zhang J. et al., 2011; Folmes et al., 2012; Hansson et al., 2012; Zhang et al., 2012; Tohyama et al., 2013). Glycolysis allows a quick supply of ATP, while the pentose phosphate pathway supplies the nucleotide precursors. ESCs have a reduced and immature mitochondrion, a more-reduced redox environment, and an increased lactate production (Yanes et al., 2010). Metabolites produced in these processes can directly influence the epigenetic and transcriptional programs of the PSCs involved in their self-renewal processes (Figure 5, reviewed in Tsogtbaatar et al., 2020).

Recent studies of both ESCs and adult stem cells suggest that the balance between self-renewal and differentiation is partly regulated by ROS which mediate the cellular redox state (Bigarella et al., 2014; Tsogtbaatar et al., 2020). ESCs, in comparison to ASCs and differentiated cells, are characterized by low ROS levels, low rate of H_2_O_2_ removal, and low threshold for H_2_O_2_-induced cytotoxicity. However, based on the biochemical normalization of the parameters, it was suggested that ESCs and differentiated cells maintain a similar intracellular redox status (Lyublinskaya et al., 2017). Similar to the differentiated cells, a prolonged exposure to high concentrations of ROS affects ESCs maintenance, leading to their differentiation or apoptosis (Guo et al., 2010). An increased activation of the oxidative phosphorylation by the inactivation of the uncoupling protein 2 (UCP2) associated with the concomitant elevation of ROS levels results in the loss of ESCs stemness, increased differentiation, and apoptosis (Zhang J. et al., 2011). In addition, the prolonged hypoxic exposure of ECSs leads to the increased levels of ROS and apoptosis. Importantly, ESCs are highly resistant to oxidative stress-induced senescence, having unique mechanisms protecting from ROS-induced senescence (Guo et al., 2010). For instance, short-term sub lethal concentration of H_2_O_2_ treatment of ESCs induces only a transient G2/M cell cycle arrest associated with the inhibition of cell adhesion and expression of cyclin D1. ESCs also maintain their genomic integrity and clonal recovery under physiological hypoxic oxygen (2%) levels (Forsyth et al., 2006). On the other hand, decreased ROS levels caused by either hypoxia that enhances glycolysis or by suppressing oxidative phosphorylation improve ESCs maintenance and proliferation, while repressing their differentiation (Mandal et al., 2011; Zhou et al., 2012). It was shown that mouse ESCs with low and high mitochondrial membrane potentials marked differences in the metabolic rates and, presumably, ROS generation. Interestingly, the ESCs with a high mitochondrial membrane potential were truly pluripotent, suggesting that the level of mitochondrial metabolism is related to ESC self-renewal (Schieke et al., 2008). Mitochondrial dynamics play a fundamental role in establishing the full pluripotency and developmental potential of PSCs. PSCs that display excessive mitochondrial fission associated with increased cytosolic Ca^(2+)^, CaMKII activity, and inactivation of β-catenin are not able to produce a live born offspring (Zhong et al., 2018). It is important to note that mitochondrial fission is also associated with an increase in mitochondrial ROS generation; the latter is involved in the proper maintenance of PSCs [see above, and (Tang et al., 2009; Jezek et al., 2018; Prieto et al., 2020)].

Endogenous ROS levels are important for ESCs maintenance in culture (Figure 5). It has been shown that the transcription factors involved in pluripotency maintenance are especially sensitive to oxidation and endogenous antioxidant enzyme activity, both regulating their functions. For example, one of the main regulators of pluripotency, the transcriptional factor Oct4, is sensitive to oxidation. During early development, processes of uterine implantation and vascularization increase oxygen exposure, resulting in the suppressed DNA binding activity of Oct4. The antioxidant enzyme thioredoxin could restore the DNA-binding activity of Oct4 by physically associating with cysteines in the POU domain of Oct4 (Guo et al., 2004). The multifunctional tumor suppressor transcription factor p53 transactivates proapoptotic genes or antioxidant genes, while in the cytoplasm, p53 induces mitochondria-dependent apoptosis. In response to endogenous ROS, NAD-dependent deacetylase sirtuin-1 (SIRT1) blocks the translocation of p53 into the nucleus and triggers the apoptosis of mouse ESCs. SIRT1 predisposes ESCs to be sensitive to ROS and inhibits p53-mediated suppression of Nanog expression. SIRT1 is also involved in the regulation of mitochondrial function upon oxidative stress of ESCs (Han et al., 2008; Ou et al., 2014). It has also been shown that Foxo1 is essential for the maintenance of mouse and human ESC pluripotency, probably through the direct activation of Oct4 and Sox2 gene expression (Zhang X. et al., 2011).

Nox4-generated ROS are involved in the proper differentiation of mouse ESCs toward the smooth muscle cell (SMC) lineage through the activation of the serum response factor (SRF), myocardin, and autocrine transforming growth factor beta (TGF-beta) (Xiao et al., 2009). Mitochondrial maturation with network expansion and enhancement of oxidative phosphorylation are involved in the differentiation of ESCs into functional cardiomyocytes (Figure 5). Disrupting ETC function prevented mitochondrial organization, compromising cardiomyocyte differentiation (Chung et al., 2007). Differentiation of cardiomyocytes from PSCs occurs more robustly and completely in a glucose-depleted culture medium, suggesting that the switch from glycolytic metabolism to oxidative phosphorylation with subsequent ROS generation is required for the proper differentiation into cardiomyocyte lineage (Tohyama et al., 2013). ROS also enhances the differentiation of human ESCs into mesendodermal cell lineage probably by the activation of the mitogen-activated protein kinase (MAPK) signaling pathways (Ji et al., 2010). The sources of ROS, the effect of ROS levels on cardiomyocyte differentiation of PSCs, and mechanism of ROS signaling during these processes are reviewed in detail elsewhere (Wei and Cong, 2018). Taken together, these studies indicate that ROS signaling regulated by cell metabolism is important for PSC maintenance and differentiation; however, many issues of this regulation remain unresolved. Future investigation of metabolic and ROS-mediated aspects of PSC biology will help to improve the differentiation and reprogramming protocols of the generation of healthy and fully potent differentiated cells for the purposes of regenerative medicine and transplantation.

### ROS Function During Cell Reprogramming to Pluripotent State

**FIGURE 6:**
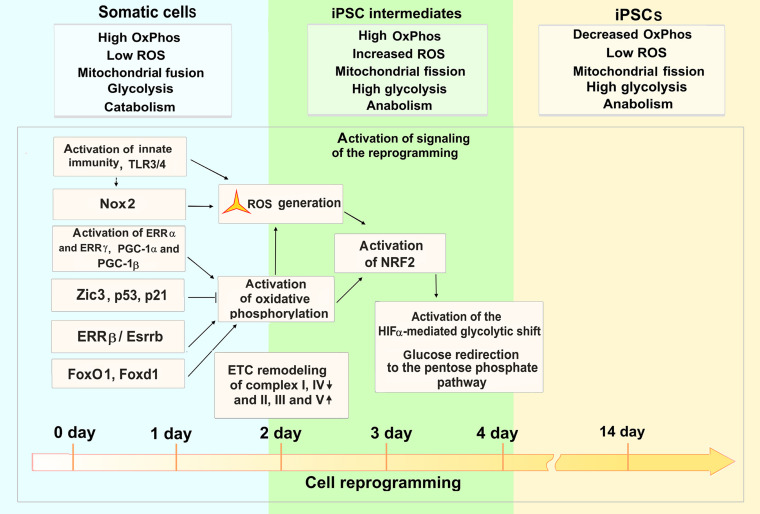
ROS functions during cell reprogramming to a pluripotent state. The OSKM-mediated cell reprogramming to pluripotency requires tight regulation of ROS levels and metabolic flux during first 4 days of the process (1–14-day time scale of mouse fibroblast reprogramming). Activation of innate immunity and Nox2 trigger ROS generation at the onset of the reprogramming. At this stage of reprogramming, estrogen-related nuclear receptors (ERRα, ERRγ, ERRβ) and forkhead box transcription factors (FoxO1, Foxd1) are involved in activation of oxidative phosphorylation (OxPhos) and ensuing ROS production. On the other hand, Zic3 and p53/p21 transcriptional regulators suppress OxPhos, activating aerobic glycolysis. The increased OxPhos and ROS lead to an activation of NRF2 transcriptional regulator of antioxidant enzymes. Further HIFa mediates metabolic shift, redirecting glucose to the pentose phosphate pathway. Metabolic features of somatic, iPSC intermediates, and fully reprogrammed iPSCs are denoted in respective boxes.

As opposed to the cell differentiation process, somatic cells can be reprogrammed into a pluripotent state by ectopic expression of defined factors—the transcription factors Oct4, Klf4, Sox2, c-Myc—being converted into induced pluripotent stem cells, iPSCs (Takahashi and Yamanaka, 2006). The developed iPSC technology opened unprecedented possibilities for PSC use in regenerative medicine, disease modeling, and therapeutic drug development. During reprogramming, cells undergo dramatic changes of a wide range of cellular characteristics, such as metabolism, mitochondrial morphology and function, and cell signaling network. These characteristics are critical for reprogramming to pluripotency, enabling indefinite self-renewal and the ability to differentiate into all cell types of an adult organism (Figure 6, reviewed in Prieto et al., 2020). The cell reprogramming to pluripotency also requires a tight regulation of the ROS levels and metabolic flux (Figure 6). Metabolic switch from oxidative phosphorylation to glycolysis seems to be critical for the initial key steps in the reprogramming process (Folmes et al., 2011). Aerobic glycolysis increases throughout the reprogramming process. Naive pluripotent stem cells (PSCs), present in the epiblast of pre-implantation blastocysts, utilize both glycolysis and oxidative phosphorylation to satisfy their metabolic needs. The key reprogramming factor Oct4 acts in the transcriptional regulation of multiple metabolic genes involved in reprogramming and pluripotency control (Kang et al., 2009). Interestingly, the ubiquitous POU-domain factor Oct1 can take over the Oct4 function during PSC differentiation in the regulation of metabolic genes (Kang et al., 2009; Shen et al., 2017). Small molecules involved in the regulation of the transition to glycolysis have been shown to mediate iPSC generation (Zhu et al., 2010).

Efficient cell reprogramming to pluripotency requires the activation of innate immunity that triggers ROS generation and signaling at the onset of the reprogramming (Lee et al., 2012; Yang C. S. et al., 2013). Nox (1–4) enzyme complex involved in generating ROS at the early stage of reprogramming and optimal levels of ROS signaling are both essential to induce pluripotency (Zhou et al., 2016). Recently, an importance of metabolic shift prior to the establishment of induced pluripotency has been shown (Kida et al., 2015). Estrogen-related nuclear receptors (ERRs) and their co-factors PGC-1α and PGC-1β are transiently induced at an early stage of the reprogramming process. ERRα or ERRγ functions are required for the burst of oxidative phosphorylation and iPSC generation in human and mouse cells, respectively. In mouse, ERRγ and PGC-1β are highly expressed in Sca1^(−)^/CD34^(−)^ *bona fide* iPSC progenitors, resulting in a burst of oxidative phosphorylation. The lack of metabolic switch induction leads to an arrest of the reprogramming process (Kida et al., 2015). Further study has shown that another ERR (ERRβ, or Esrrb) and Zic3 transcription factors synergistically enhance the reprogramming efficiency by regulating cellular metabolic pathways (Figure 6). The two factors cooperatively activate the glycolytic metabolism and moreover, Zic3 represses, whereas Esrrb activates oxidative phosphorylation which is also critical for the conversion of primed PSCs, present in the epiblast after implantation, into the naive state, a characteristic for a pre-implantation epiblast (Sone et al., 2017). It has also been shown that NRF2 is critical for metabolic reprogramming. An early burst in oxidative phosphorylation and ROS generation induces the activation of NRF2 which, in turn, initiates the HIFa-mediated glycolytic shift and, probably, modulates glucose redirection to the pentose phosphate pathway (Hawkins et al., 2016). Forkhead box transcription factors, Foxd1 and Foxo1, may also be involved in oxidative burst regulation during the reprogramming as their genetic knockdown suppresses the generation of iPSCs (Koga et al., 2014). These factors are crucial for the regulation of ROS and cellular metabolism (Yeo et al., 2013). Quantitative proteomics analysis showed highly coordinated changes in the expression of functionally related proteins during the first and last 3 days of the reprogramming, that includes changes in the stoichiometry of ETC complexes during the early and intermediate stages (Hansson et al., 2012). In particular, decreased expressions of complexes I and IV, as well as increased expressions of complexes II, III, and V subunits of ETC occur at these stages. The reduced expression of complex I may indicate an increased ROS production by the mitochondria during the onset of the reprogramming. Importantly, it was shown recently that the mitochondrial genome and functions are well preserved throughout the processes of reprogramming the peripheral blood mononuclear cells to iPSCs, as well as on the subsequent differentiation of the iPSCs into functional cerebral organoids (Duong et al., 2021).

It has been shown that at the mid and late stages of reprogramming, ROS generation is increased, having some damaging effect for the cell genome. In this regard, general antioxidants reduce the genome instability during reprogramming, suggesting that antioxidants supplementation might enhance the quality and safety of human iPSCs (Ji et al., 2014). ROS stress defense mechanisms and mitochondrial biogenesis are similar in human iPSCs and ESCs (Armstrong et al., 2010). In immortalized somatic cells or cancer cells, the metabolism is reverted to aerobic glycolysis associated with suppressed oxidative phosphorylation and reduced ROS levels, possibly serving as barriers for the reprogramming of these cells to a pluripotent state (Vazquez-Martin et al., 2012; Skvortsova et al., 2018). Importantly it was shown that iPSCs generated from aged donors (A-iPSCs) are unable to suppress oxidative phosphorylation. In contrast to young donors-derived ESCs and iPSCs, specific glucose transporter 3 (GLUT3) is significantly suppressed in A-iPSCs, resulting in an impaired glucose uptake. In this regard, A-iPSCs generate increased ROS levels, leading to the elevation of ROS-scavenging glutathione and DNA damage response. Activation of oxidative phosphorylation in young donor iPSCs recapitulates these phenotypes of A-iPSCs (Zhang et al., 2017).

### Example of Redox Regulation of Protein Function: Non-histone High Mobility Group Proteins

**FIGURE 7:**
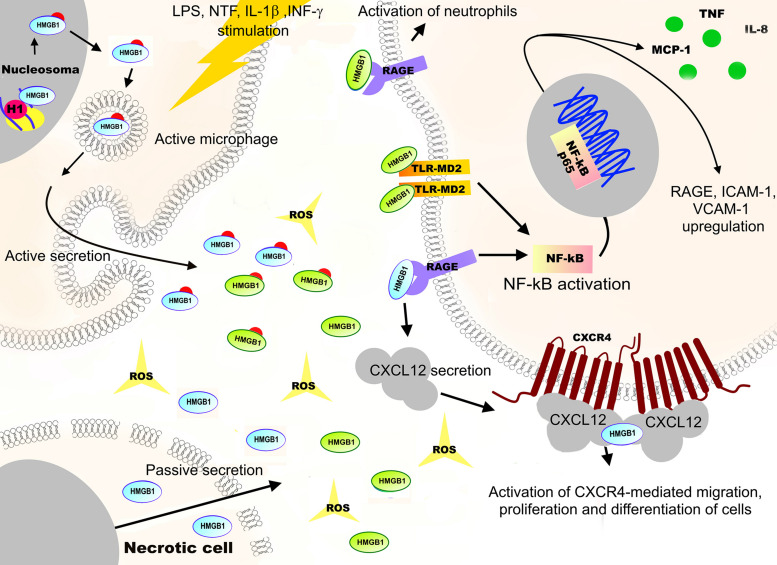
Functions and redox regulation of the high mobility group protein B1 (HMGB1). The non-histone high mobility group box 1 (HMGB1) protein is one of the examples of redox-dependent proteins. Reduced nuclear HMGB1 is a regulator of chromatin structure, involved in DNA replication, recombination, transcription, telomere maintenance, and DNA repair. Hyperacetylation of HMGB1 nuclear localization sequences (NLS) by oxidative stress, lipopolysaccharide (LPS), or several neurotrophic factors (NTF) stimulation can lead to translocation of the protein to the cytoplasm. The reduced form of cytoplasmic HMGB1 binds with the beclin1 protein, promoting the autophagosome formation while blocking apoptosis. The oxidation of HMGB1 leads to its translocation into the cytoplasm that is associated with suppression of the NRF2 expression and downstream antioxidant genes, leading to apoptosis. In addition, if cell integrity is damaged, HMGB1 is released into extracellular space where, upon ROS exposure, it is oxidized at Cys23, Cys45, and Cys106. Depending on the oxidation state, oxidized HMGB1 acts as a signaling molecule that initiates cell migration, cellular immune response, and synthesis of anti-inflammatory cytokines. These effects are achieved through activation of MAPKs, NF-κB, and phosphoinositide 3-kinase/AKT signaling pathways, as well as through interaction with a receptor for advanced glycation endproducts (RAGE), Toll-like receptors (TLR2, TLR4), the TREM1 myeloid cell trigger receptor, and the CD24 differentiation receptor.

A large amount of literature data indicates that intra- and extracellular redox potential affects the structure of proteins and the secretion and function of cytokines. One of the best examples of redox-dependent proteins is the non-histone high mobility group box 1 (HMGB1) protein which is widespread in the chromatin. HMGB1 is involved in a variety of cellular processes, depending on its modification status and redox state of the Cys23, Cys45, and Cys106 residues (reviewed in Chikhirzhina et al., 2020). The close spatial localization of these Cys residues is required for HMGB1 DNA binding, indicating the importance of the redox-regulated conformational changes of the protein for its functional affinity (Figure 7; Ohndorf et al., 1999; Chikhirzhina et al., 2020).

HMGB1 protein is a regulator of chromatin structure. In the nucleus, it functions as a DNA chaperone. HMGB1 induces the binding of the chromatin remodeling complex to the nucleosome and facilitates the movement of the nucleosome along the DNA. This occurs by the chromatin unwinding due to the displacement of histone H1 from the nucleosome by HMGB1, further allowing the recruitment of the transcription factors to DNA (Verrijdt et al., 2002). This function of HMGB1 as a DNA chaperone allows an involvement of the protein in a wide range of nuclear processes such as DNA replication, recombination, transcription, telomere maintenance, and DNA repair (Yuan et al., 2004). In addition, in the nucleus, HMGB1 protein interacts with tumor suppressors, such as p53 (Jayaraman et al., 1998; Rowell et al., 2012), members of the REL transcription factor family (RELA/P65, c-REL, RELB, P50/NF-κB1, and p52/NF-B2 (Agresti et al., 2003), and cyclin-dependent kinases like CDK2 (Neganova et al., 2011).

It has been shown that an increase in the intracellular H_2_O_2_ concentration leads to a partial oxidation of HMGB1 by forming a Cys23–Cys45 disulfide bridge and reduced Cys106, resulting in a complete loss of HMGB1 nuclear functions (Hoppe et al., 2006; Polanska et al., 2014). Indiscriminate covalent modification of HMGB1 thiols causes the inhibition of protein binding to different types of DNA (Sheflin et al., 1993; Stros et al., 1994) and to interact with histone H1 (Kohlstaedt and Cole, 1994). It has been shown that oxidation of HMGB1 leads to its translocation to the cytoplasm, associated with the suppression of the NRF2 expression and downstream antioxidant genes, and results in apoptosis (Mou et al., 2018). ROS-induced HMGB1 translocation to the cytosol also causes autophagy in cultured fibroblasts (Tang et al., 2010). The molecular mechanisms of HMGB1 translocation from the nucleus to the cytosol are directly related to the H_2_O_2_-induced calcium homeostasis disturbance (Zhao et al., 2017). An increase in the level of intracellular calcium leads to the increased translocation from the cytoplasm to the nucleus of two calcium-dependent enzymes, PKCα and CaMKIV, which promotes the phosphorylation of HMGB1. This, in turn, leads to HMGB1 translocation to the cytoplasm and its subsequent release into the extracellular space. In addition, hyperacetylation of HMGB1 nuclear localization sequences (NLS) by oxidative stress can also lead to the translocation of the protein to the cytoplasm (Lu et al., 2014).

The reduced form of cytoplasmic HMGB1 binds the beclin1 protein, promoting autophagosome formation, while blocking apoptosis (Tang et al., 2011). However, ROS-induced partial oxidation of HMGB1 (Cys23–Cys45 disulfide bridges and reduced Cys106) leads to the activation of caspase-3 and caspase-9, inducing the mitochondrial apoptosis pathway. If cell integrity is lost, HMGB1 is released into the extracellular space and, upon ROS exposure, becomes oxidized at Cys23, Cys45, and Cys106 (Tang et al., 2011). Depending on the oxidation state, HMGB1 interacts with a number of transmembrane receptors, including the receptor of advanced glycation endproducts (RAGE) (Tian et al., 2007), Toll-like receptors (TLR2, TLR4) (Venereau et al., 2012; Yang et al., 2015; Raucci et al., 2019), the TREM1 myeloid cell trigger receptor (El Mezayen et al., 2007), and the CD24 differentiation receptor (Chen et al., 2009). It is well established that the interaction of the reduced form of HMGB1 with RAGE stimulates the production of the chemokine (C-X-C motif) ligand 12 (CXCL12). The interaction of HMGB1 with the CXCL12, in turn, leads to the interaction of the protein with the CXC chemokine receptor type 4 (CXCR4 receptor), resulting in the activation of cell migration, proliferation, and differentiation during tissue healing and regeneration (Figure 7; Lee et al., 2018; Raucci et al., 2019). At the same time, the interaction of RAGE with the disulfide form of HMGB1 leads to the activation of neutrophils and the formation of their extracellular traps, which is important in thrombotic inflammatory processes (Stark et al., 2016). Disulfide form of HMGB1 also interacts with the Toll-like receptor 4/myeloid differentiation factor 2 complex (TLR4-MD2), which leads to a release of inflammatory and angiogenic factors through the activation of the transcription factor NF-kB (Raucci et al., 2019). Oxidized HMGB1 acts as a signaling molecule that initiates cell migration, cellular immune response, and synthesis of anti-inflammatory cytokines by activating the MAPKs, NF-kB, and phosphoinositide 3-kinase/AKT signaling pathways. In conclusion, the example with HMGB1 shows how a reversible formation of an intramolecular disulfide bond in a protein can generate a highly sensitive mechanism of redox-dependent control of gene expression, DNA replication, and repair.

## Conclusion

The extensive studies in the last two decades have shown that ROS, along with their damaging activities, mediate many important signaling functions in different cellular and developmental processes of metazoans. The level and sources of intracellular ROS are critically important for its physiological function. In this review, we have discussed the main discoveries regarding the physiological signaling functions of ROS in the cell and developmental processes of the invertebrate *Drosophila* and of mammalians including hematopoiesis, adult stem cells, tissue regeneration, and pluripotent stem cell biology, iPSCs generation, as well as systemic cell non-autonomous functions of ROS. *Drosophila* remains the most advanced organism to identify and to study gene function and signaling pathways in various evolutionary conserved cellular and developmental processes, yet many ROS functions identified in flies remained to be investigated in mammals. The insight into mitochondrial interaction with intracellular signaling pathways and transcriptional regulation via the ROS second messenger activity will further help to efficiently generate high quality stem cells and derive thereof differentiated cells for the purposes of regenerative medicine. Importantly, the listed advances in ROS biology research indicate that the precise manipulation of ROS levels and of their sources with the help of molecular drugs will eventually help tackle a wide range of human diseases associated with an altered redox system, including cancer and autoimmune pathologies.

## Author Contributions

SS: conceptualization, design, and writing of the manuscript. TS, AK, and AT: writing of the manuscript. SS, TS, and AK: design and preparation of the figures. All authors contributed to the article and approved the submitted version.

## Conflict of Interest

The authors declare that the research was conducted in the absence of any commercial or financial relationships that could be construed as a potential conflict of interest.

## Publisher’s Note

All claims expressed in this article are solely those of the authors and do not necessarily represent those of their affiliated organizations, or those of the publisher, the editors and the reviewers. Any product that may be evaluated in this article, or claim that may be made by its manufacturer, is not guaranteed or endorsed by the publisher.

## Footnotes

1
https://doi.org/10.1101/2021.01.06.425530
